# Dictamnine Inhibits the Adhesion to and Invasion of Uropathogenic *Escherichia Coli* (UPEC) to Urothelial Cells

**DOI:** 10.3390/molecules27010272

**Published:** 2022-01-02

**Authors:** Wenbo Yang, Peng Liu, Ying Chen, Qingyu Lv, Zhongtian Wang, Wenhua Huang, Hua Jiang, Yuling Zheng, Yongqiang Jiang, Liping Sun

**Affiliations:** 1 College of Chinese Medicine, Changchun University of Chinese Medicine, Changchun 130117, China; shiguang20140702@163.com (W.Y.); 18088603077@163.com (Z.W.); 2 State Key Laboratory of Pathogen and Biosecurity, Institute of Microbiology and Epidemiology, Academy of Military Medical Sciences, Beijing 100071, China; ammsliupeng@163.com (P.L.); lvqingyu2004@126.com (Q.L.); huangwh1993@163.com (W.H.); jhua76@126.com (H.J.); zhengyuling@sina.com (Y.Z.); 3 School of Light Industry, Beijing Technology and Business University (BTBU), Beijing 100048, China; chenying@btbu.edu.cn

**Keywords:** dictamnine, urinary tract infection, UPEC, adhesion invasion, fimbriae

## Abstract

Uropathogenic *Escherichia coli* (UPEC) is the most common pathogenic bacteria associated with urinary tract infection (UTI). UPEC can cause UTI by adhering to and invading uroepithelial cells. Fimbriae is the most important virulence factor of UPEC, and a potentially promising target in developing novel antibacterial treatments. In this study, the antibacterial properties and effects of the compound dictamnine, extracted from the traditional Chinese medicine Cortex Dictamni, on the bacterial morphology, cell adhesion, and invasion of UPEC were studied. Dictamnine exhibited no obvious antibacterial activity against UPEC, but significantly impeded the ability of UPEC to adhere to and invade uroepithelial cells. RT-qPCR analysis showed that treatment downregulated the expression of type 1 fimbriae, P fimbriae, and curli fimbriae adhesion genes, and also downregulated adhesion-related receptor genes of uroepithelial cells. Transmission electron microscopy showed that dictamnine destroyed the structure of the fimbriae and the surface of the bacteria became smooth. These results suggest that dictamnine may help to prevent UTI by simultaneously targeting UPEC fimbriae and urothelial adhesin receptors, and may have a potential use as a new anti-UPEC drug.

## 1. Introduction

Uropathogenic *Escherichia coli* is the main causal agent of urinary tract infections (UTIs) [1]. One hundred fifty million people suffer from UTIs every year worldwide [2], with women having a higher risk of contracting an UTI [3]. In female UTI patients, UPEC is responsible for 80% of all cases [4]. UTIs have caused great economic and health burdens; in the United States alone, the annual medical expenses and time costs associated with urinary tract infections exceeds 3.5 billion USD [5], and the urinary tract is the most important source of *E. coli* bacteremia [6]. The high incidence and incurred harm of urinary tract infections highlights the importance of finding new treatments.

UPEC adhesion to urothelial cells is a prerequisite for initiating a urinary tract infection [5]. Fimbriae are the most critical adhesion factor, and main virulence factor, of UPEC. The expression of Type 1 fimbriae, P fimbriae, and Curli fimbriae can promote UPEC adhesion to and invasion of host cells [7,8]. Type 1 fimbriae are among the most important virulence factors involved in the contraction of UTIs [9]. Expression of P fimbriae is closely related to acute pyelonephritis [10], and Curli fimbriae participate in biofilm formation and are involved in the adhesion of UPEC to the bladder [8]. Integrinsα3, β1, and uroplakins are the main host cell receptors to which UPEC adhere [11,12]. Type 1 fimbriae mediate bacterial adhesion to and invasion of bladder epithelial cells [7]. When a urinary tract infection occurs, UPEC adhere to urothelial cells through type 1 fimbriae, which bind to urothelial cell integrin and uroplakins receptors, allowing UPEC to invade and colonize the bladder and form intracellular bacterial communities (IBCs) [13]. After bladder invasion, UPEC moves up the ureter through the function of P fimbriae and causes acute pyelonephritis. Simultaneously, UPEC produce adhesion factors that promote biofilm formation [14]. Biofilms are bacterial communities intertwined in a matrix of polysaccharides, proteins, and nucleic acids [15], which can protect bacteria from host immune responses and antimicrobial treatments [16]. UPEC adhesion factors act to form a biofilm and promote the survival of UPEC in the urinary tract [17], increasing the resistance of UPEC to antimicrobial treatment [18]. Therefore, the adhesion of fimbriae to the urothelium plays a crucial role in UPEC infection of the urinary tract and the antibiotic resistance of UPEC in UTIs.

**Figure 1:**
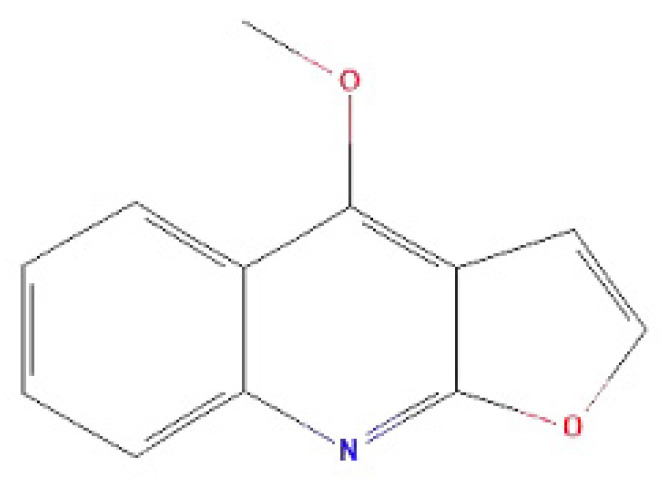
Chemical structure of dictamnine.

Antibiotics are a routinely used treatment for UTIs [19], despite the fact that antibiotic resistance is increasing worldwide [20]. Routine use of antibiotics can produce resistant microbial strains that can persist for up to six months [21]. Therefore, developing non-antibiotic-based treatments is of paramount importance. Cortex Dictamni is a traditional Chinese medicine that is typically used to treat skin and urinary system diseases. Dictamnine is an alkaloid isolated from Cortex Dictamni that exhibits antibacterial, antifungal, and anticancer properties [22,23,24]. Dictamnine is the most abundant component of Cortex Dictamni and its structure has been clarified [25,26]. However, there are no reports of its use in treatment of UTIs. In this study, we examined the antibacterial effects of dictamnine (Figure 1) and its effects on UPEC adhesion to and invasion of T24 cells. RT-qPCR analysis was used to evaluate the effect of dictamnine on the expression of fimbriae genes. Transmission electron microscopy was used to observe the effects of dictamnine on the morphology of UPEC fimbriae. This study is the first to determine the effects of dictamnine on UPEC fimbriae, and subsequent effects on UPEC adhesion to and invasion of uroepithelial cells, highlighting its potential use as an effective, non-antibacterial-based treatment for urinary tract infections.

## 2. Results

### 2.1. Cytotoxicity of Dictamnine to Urothelial Cells

**Figure 2:**
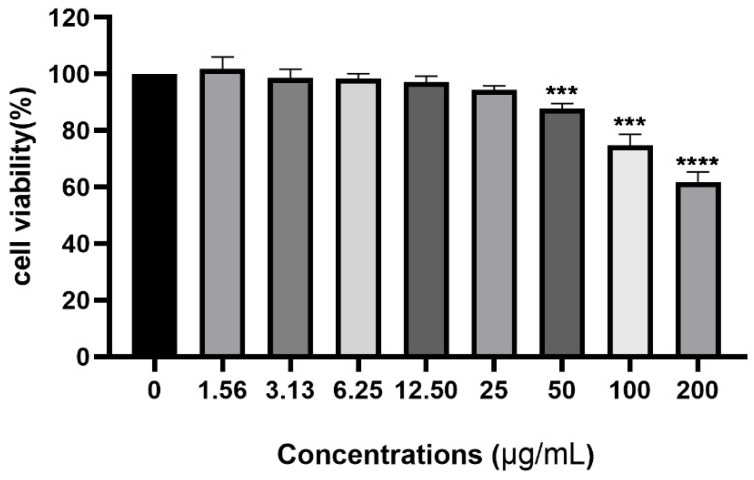
Influence of dictamnine, at concentrations ranging from 0 to 200 µg/mL, on the cell viability (CCK-8 assay) of T24 cells after 24 h incubation. Values represent the mean ± SD from three independent experiments containing 18 technical replicates. *** *p* < 0.001, **** *p* < 0.0001.

To determine the potentially detrimental effects of dictamnine on host cells, we used the CCK-8 method to study its cytotoxicity to the urothelial cell line T24. As shown in Figure 2, the concentration of dictamnine ranged from 1.562 µg/mL to 200 µg/mL. After a 24 h treatment, the viability of cells treated with over 25 µg/mL of dictamnine significantly decreased (*p* < 0.05) compared with the control group. Dictamnine treatment at concentrations below 25 µg/mL produced no obvious cytotoxicity and the cell viability was not statistically significant from the control group. Therefore, we used concentrations of dictamnine below 25 µg/mL for subsequent experiments.

### 2.2. UPEC Growth in the Presence of Dictamnine

**Figure 3:**
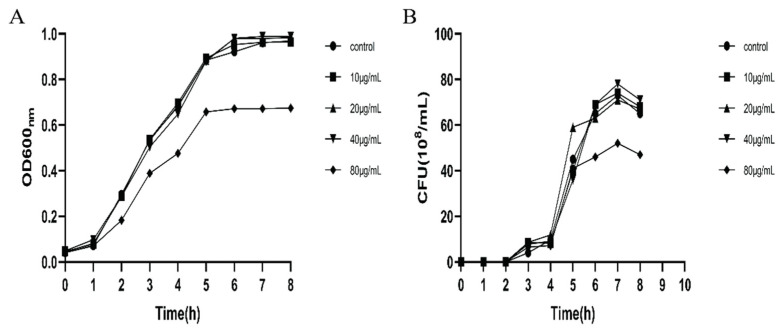
Effects of dictamnine on the in vitro growth of UPEC307. (**A**) Absorbance of bacteria at 600 nm at different time points; (**B**) number of viable bacteria at different time points. CFU: colony-forming unit.

The growth curve of UPEC in the presence of different concentrations of dictamnine is shown in Figure 3. The bacterial density and number of colonies (CFU) show that dictamnine has weak antibacterial activity against UPEC. At a concentration of 80 µg/mL, dictamnine has an inhibitory effect on the growth of UPEC. When the concentration of dictamnine is lower than 20 µg/mL, it has little effect on the growth of UPEC.

### 2.3. Dictamnine Reduces UPEC Adhesion to and Invasion of Urothelial Cells

**Figure 4:**
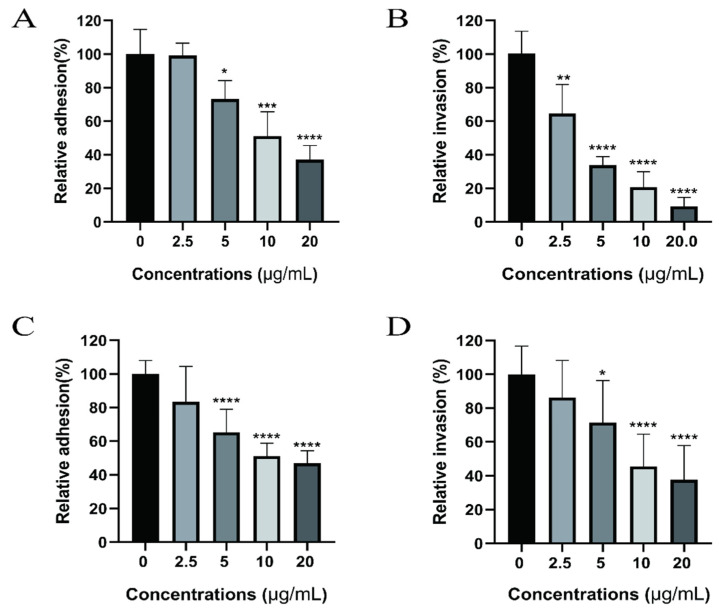
Effect of dictamnine on cellular adhesion and invasion by UPEC. The relative adhesion rate (**A**) and relative invasion rate (**B**) of UPEC to T24 cells after co-incubation of UPEC with T24 cells for 2 h. The relative adhesion rate (**C**) and relative invasion rate (**D**) of UPEC to T24 cells after 2 h of pre-incubation of dictamnine with T24 cells. Values represent the mean ± SD from three independent experiments with three technical replicates. * *p* < 0.05, ** *p* < 0.01, *** *p* < 0.001, **** *p* < 0.0001.

Adhesion to and invasion of UPEC into urothelial cells is the initial stage of urinary tract infection. In this study we determined the effect of dictamnine on the adhesion and invasion ability of UPEC by allowing UPEC to infect T24 cells and treating infected cells with different concentrations of dictamnine ranging from 2.5 to 20 µg/mL, for 2 h; the results are shown in Figure 4. The results show that dictamnine treatment above 5 µg/mL can reduce UPEC adherence to T24 cells (Figure 4A). After gentamicin was added to kill bacteria on the cell surface, it was found that dictamnine treatment also reduced the invasion of T24 cells by UPEC (Figure 4B). Additionally, we explored whether dictamnine could prevent the occurrence of urinary tract infections. T24 cells were pretreated with different concentrations of dictamnine for 2 h, after which UPEC was added to initiate cell infection; results are shown in Figure 4C,D. Treatment with dictamnine at concentrations above 5 µg/mL can reduce the adhesion and invasion of UPEC to T24 cells, and can potentially help prevent urinary tract infections.

### 2.4. Effect of Dictamnine on UPEC and Urothelial Cells’ Gene Expression

**Figure 5:**
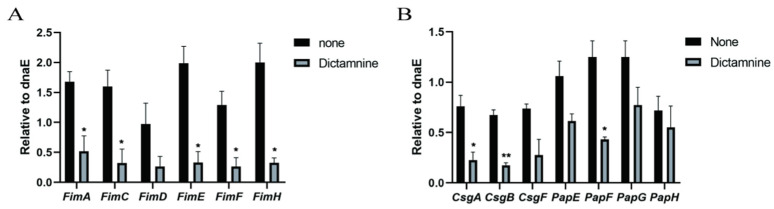
Effects of dictamnine on fimbriae production. Evaluation of expression levels of fimbriae-associated genes by RT-qPCR: (**A**) type 1 fimbriae, (**B**) curli fimbriae and P fimbriae. * *p* < 0.05, ** *p* < 0.01.

**Figure 6:**
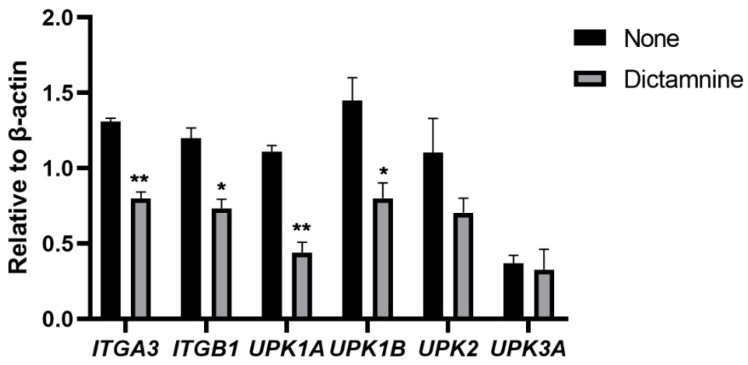
Expression of integrin and uroplakin genes in T24 bladder epithelial cells treated with dictamnine determined by RT-qPCR. * *p* < 0.05, ** *p* < 0.01.

Next, we explored potential reasons why dictamnine reduces UPEC adhesion and invasion. Adhesion and invasion occur due to the interaction between UPEC fimbriae and urothelial cell adhesin receptors, as shown in Figure 5. The fimbriae of UPEC mainly include type 1 fimbriae, P fimbriae, and Curli fimbriae. After the addition of dictamnine to UPEC cells, the expression levels of adhesion genes associated with type 1 fimbriae and Curli fimbria, which are involved in lower urinary tract infections, were all significantly reduced (Figure 5A,B). Interestingly, although P fimbriae are mainly involved in the development of acute pyelonephritis, dictamnine also reduces the expression of some P fimbriae genes (Figure 5B). Urothelial cell adhesion receptors mainly include integrin and uroplakin receptors. Dictamnine treatment reduced the expression levels of integrin receptor α3 and β1 genes. Among uroplakin genes, the expression of UPK1A was significantly reduced, while UPK3A did not decrease significantly (Figure 6). These results indicate that dictamnine may act on both UPEC fimbriae and T24 cell adhesion receptors.

### 2.5. Inhibitory Effect of Dictamnine on UPEC Fimbriae

**Figure 7:**
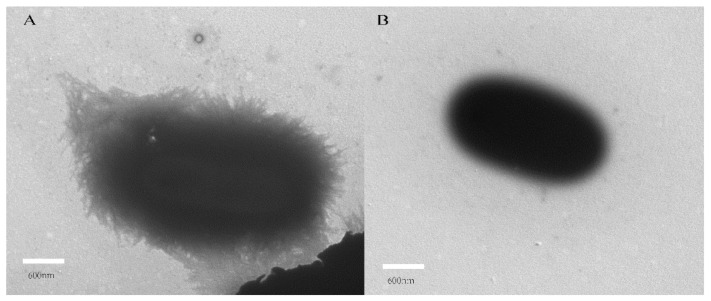
Transmission electron microscopy images of growing UPEC fimbriae. (**A**)Untreated UPEC fimbriae and (**B**) dictamnine-treated UPEC fimbriae.

Fimbriae are the main adhesion structure of UPEC. After treatment with dictamnine, it can be seen through TEM that the edges of UPEC cells become smooth and lack the hair-like structures on the cell surface that can be seen in untreated UPEC (Figure 7). This demonstrates that dictamnine caused the loss of fimbriae on the surface of UPEC cells, and thus may be a potential inhibitor of UPEC fimbriae. The ability of dictamnine to reduce the adhesion and invasion ability of UPEC may be related to its effects on UPEC fimbriae.

## 3. Discussion

Currently, antibiotics are still widely used in the treatment of UTI. However, the increasing development of antibiotic-resistant bacterial strains has forced humans to urgently seek new alternative treatment methods, among which traditional herbal medicine has attracted much attention. Compared with antibiotics, traditional herbal medicine employs an alternative antibacterial mechanism. Early studies have shown that traditional herbs and their extracts can protect urothelial cells from the adhesion and invasion of UPEC [27,28,29]. In the current study, we used an in vitro model of bladder epithelial cell infection by UPEC to evaluate the role of dictamnine in treating UTI, and demonstrated that dictamnine not only diminished UPEC fimbriae (a critical virulence factor) and reduced UPEC colonization of T23 cells, but also was able to protect urothelial cells from UPEC infection.

Our research found that treatment with dictamnine at concentrations below 25 µg/mL did not inhibit the growth of UPEC, showing that dictamnine had no direct antibacterial effect on UPEC. To explore the potential mechanisms underlying the effects of dictamnine, RT-qPCR was used to analyze the effects of dictamnine on expression of fimbriae coding genes. We speculate that the inhibition of type 1 fimbriae, P fimbriae, and Curli fimbriae genes by dictamnine is one explanation for the inhibition of fimbriae observed. *FimH* and *PapG* are the adhesins at the tip of the fimbriae, which mediate the adhesion and invasion of UPEC into host cells [30]. Decreased expression of *FimH* and *PapG* may directly reduce the adhesion and invasion of the bacteria to host cells. *FimA*, *FimC*, and *FimD* are subunits of the type 1 fimbriae structure [31]; *CsgA* is the main subunit of Curli fimbriae; and *CsgB* and *CsgF* promote the formation and secretion of *CsgA* [32,33]. The decreased expression levels of these subunit genes may cause the loss of fimbriae. However, the exact mechanism by which dictamnine inhibits the expression of fimbriae genes is still unclear.

*UPIa* (*UPK1a*) and *UPIIIa* (*UPK3a*) are suitable ligands for type 1 fimbriae adhesin [34], among which *UPIa* is the receptor for *FimH* adhesin [35]. Our research shows that dictamnine reduces the expression of *UPIa* in bladder epithelial cells, which may be one of the mechanisms for reducing UPEC adhesion and invasion. Phagocytosis plays an important role in the host’s resistance to bacterial infections. However, UPEC can destroy the antibiological pathway of macrophages in the body to increase the amounts of intracellular bacteria [36]. *UPIIIa* may be directly or indirectly combined with *UPIa* to participate in signal transduction to activate the phagocytosis of macrophages [37], but the results of studies have shown that dictamnine does not change the expression of *UPIIIa*. *β1* and *α3* integrins can bind directly to *FimH*. Dictamnine reduces the expression of *β1* and *α3* integrins. Based on these findings, we believe that dictamnine can increase the defense capabilities of urothelial cells and protect cells from UPEC adhesion and invasion.

## 4. Materials and Methods

### 4.1. Bacterial Strains, Cell Culture, and Chemicals

The clinically isolated strain UPEC307 was collected from a urinary tract infection patient and used in all infection experiments. UPEC307 was cultured in Luria Bertani (LB) broth at 37 °C with shaking at 180 r/min until the mid-log phase, at which point bacteria were collected and the culture concentration was determined by spectrophotometry.

T24 cells (HTB-4) were purchased from ATCC and grown in a DMEM (Sigma, St. Louis, MO, USA) medium supplemented with 10% fetal bovine serum (Procell, Wuhan, China) and 1% penicillin/streptomycin (TransGen Biotech, Beijing, China). Cells were cultured at 37 °C under 5% CO_2_. Dictamnine was purchased from Chengdu Herbpurify Co., Ltd. (Chengdu, China), dissolved in dimethyl sulfoxide (DMSO, Solarbio Life Sciences, Beijing, China), and filtered with a 0.2 µm filter (Pall, New York, NY, USA) to remove bacteria.

### 4.2. Cell Viability Assay

Cell Counting Kit-8 was purchased from MCE (Monmouth Junction, NJ, USA). T24 cells were seeded in a 96-well plate at 5 × 103 cells per well (100 µL) and precultured in a 37 °C incubator for 20 h. Cells were then treated with different concentrations of dictamnine solution (0, 1.56, 3.125, 6.25, 12.50, 25, 50, 100, and 200 µg/mL), added into each well, and incubated for 24 h. After 24 h, 10 µL of CCK-8 solution was added to each well, and cells were incubated for 3 h at 37 °C; then the absorbance at 450 nm was measured with a microplate reader (Thermo Fisher Scientific, Waltham, MA, USA).

### 4.3. Antibacterial Activity Assays

UPEC307 was cultured overnight in LB broth at 37 °C with shaking at 180 r/min. 50 µL of bacterial solution was inoculated into 5 mL of sterile LB broth, different concentrations (0, 10, 20, 40, and 80 µg/mL) of dictamnine in DMSO were added to obtain the final concentrations, and cells were returned to the described culture conditions. The growth curve was measured with a microplate reader to evaluate antibacterial activity.

### 4.4. Adhesion and Invasion Assays

In adhesion assays, T24 cells were seeded in a 24-well plate with serum-free DMEM medium lacking antibodies and cultured at 37 °C under 5% CO_2_, until each well contained 2 × 10^5^ cells. Cells were washed with phosphate-buffered saline (PBS), pretreated with different concentrations of dictamnine solution (0, 2.5, 5, 10, and 20 µg/mL) for 2 h, then added to bacteria cultures at a ratio of 100:1 to initiate cell T24 cell infection; additional treatments involved the simultaneous addition of different concentrations of dictamnine solution (0, 2.5, 5, 10, and 20 µg/mL) and bacteria culture together. Two hours after allowing bacteria to infect T24 cells, the cells were washed with PBS to remove bacteria that had not adhered to the cell surface. Cells were lysed with 1% Soponin, and eluted bacteria were diluted and inoculated onto LB agar plates. After incubating overnight at 37 °C, the number of bacteria adhering to T24 cells was calculated based on the number of colonies that grew on the plates, using the following formula: relative adhesion rate = (CFU of each group/CFU in Control) × 100%. The treatments used in the invasion assays were the same as those used in the adhesion assays. After bacteria were allowed to infect T24 cells for 2 h, gentamicin (100 µg/mL) treatment for 30 min was used to kill the extracellular bacteria, after which cells were washed with PBS and lysed using the method described for the adhesion assays; then the number of bacteria was calculated and the relative invasion rate calculated.

### 4.5. Total RNA Extraction and RT-qPCR Analysis

We treated the bacteria with 10 µg/mL dictamnine and treated the cells according to the method of adhesion assay for 2 h. The RNeasy Mini Kit (Qiagen, Hilden, Duesseldorf, Germany) was used to extract total RNA from bacterial and T24 cells according to the manufacturer’s protocol. A Nanodrop spectrophotometer (Thermo Fisher Scientific, Waltham, MA, USA) was used to determine the concentration and purity of extracted RNA. We removed the genomic DNA and performed cDNA synthesis using a Mighty Script First Strand cDNA Synthesis Master Mix (Sangon Biotech, Shanghai, China). SYBR@Green Master Mix (Applied Biosystems, Waltham, MA, USA) was used for RT-qPCR. The ΔCT method was used to calculate the threshold cycle (ΔCT) value of each gene to determine the relative mRNA level. The dnaE gene according to Dai [38] and human housekeeping gene β-actin were used as reference genes for bacterial and T24 cells, respectively. The PCR primers used in this study are shown in Table 1. The analysis of samples was based on at least three independent experiments.

### 4.6. Transmission Electron Microscopy (TEM) Analysis of Fimbriae

We treated the bacteria with 10 µg/mL dictamnine at 37 °C with shaking at 180 r/min for 2 h. Bacterial cultures were mixed 1:1 (*v*/*v*) with a solution of 2.5% glutaraldehyde and incubated at 4 °C for 2 h, then dropped onto a 200-mesh carbon–Formvar copper grid, negatively stained with 3% molybdate (PH 7.0) for 35 s, and used for transmission electron microscopy (Hitachi-7700, Tokyo, Tokyo Met, Japan) to observe the presence of fimbriae.

### 4.7. Statistical Analysis

All statistical data were analyzed using GraphPad Prism (version 8; La Jolla, CA, USA). Unpaired two-tailed Student’s *t*-tests or Mann–Whitney U tests were used as appropriate. Differences of *p* < 0.05 were considered statistically significant.

## 5. Conclusions

In conclusion, our research suggests that dictamnine is a dose-dependent inhibitor of UPEC adhesion and invasion of T24 cells that acts by reducing the number of bacterial fimbriae and T24 cell adhesin receptors and has potential applications in the prevention and treatment of UTIs. Part of the inhibitory effect of dictamnine on fimbriae can be attributed to the downregulation of UPEC fimbriae-encoding genes and T24 urothelial receptor genes in response to dictamnine treatment. Because fimbriae and urothelial adhesin receptors play an important role in the adhesion and invasion of UPEC, dictamnine may prove to be a promising drug for the treatment of UPEC infection, which will contribute to the development of alternative, non-antibiotic-based strategies for the treatment of UTIs.

## Figures and Tables

**Table 1:** Primers used in this study.

Gene	Forward Primer	Reverse Primer
dnaE	GCTCGCGGGCTTGCTAT	TCGGTTTAAAAGCTGGTCATCA
β-actin	GCGCGGCTACAGCTTCA	TCCTTAATGTCACGCACGATTT
FimA	ACTCTGGCAATCGTTGTTCTGTCG	ATCAACAGAGCCTGCATCAACTGC
FimC	GCCCACTGAAGAACGGATTTT	AGTCCGGTCAGCCCTTT
FimD	CGCGCGTTGGGATAAAACT	CAAACGGCAGCGGCTTA
FimE	GCGGGAGTCGGCTTTCTC	ATACCGGCATCGCGAATAAT
FimF	TGTGGCTGGCGGTGAGT	CCGCGGATAGTAATCGTGCTA
FimH	GTACCAGCCCGCCGTAATCAT	GTCGATGGCGGGTCAAGTAT
CsgA	GCGGTAATGGTGCAGATGTTG	CGTTGGGTCAGATCGATTGA
CsgB	CGGCAGGGAGGCTCAAA	CCCGGTTGCTACTACCTTCTTG
CsgF	CCGCGATGGTCAATTGC	GGTCGAGGTTTGTCCGGTTT
PapE	CCGCCGAGGTAACCAAAAA	GCAAGCGCGCCAGAGA
PapF	CCCGTGAAGAGCTGCGTAA	TCTCGGCGCCAGCAA
PapG	TTCGCGGCCAGGATCTC	AGCCTTACGTTTCGCTTCCA
PapH	GGTCTGGCGGCAATCG	CAGGAATTTACCCCCGAGGAT
ITGA3	AAGGGACCTTCAGGTGCA	TGTAGCCGGTGATTTACCAT
ITGB1	GAAGGGTTGCCCTCCAGA	GCTTGAGCTTCTCTGCTGTT
UPK1A	TGTCCAACCCATCCCTGATC	GGTGTCCGCGCTGTAGAAG
UPK1B	CAGCCTCTACCCACTGCTTGA	GATCCAGGCAGCCCCATAG
UPK2	TCCAGCAGAGAGATCCCAATG	CCAGCCCAATGGATTCCA
UPK3A	TTCGGCTCGGCTGTGAAC	GTTGGTGGCGAAAGTCACACT

## Data Availability

Samples of the compounds are available from the authors.

## Sample Availability

Samples of the compounds dictamnine are available from the authors.
